# The Need to Implement Health Technology Assessment in Polish Hospitals—A Survey of 50 Hospital Managers

**DOI:** 10.3390/ijerph19148855

**Published:** 2022-07-21

**Authors:** Michał M. Farkowski, Krzysztof Lach, Malwina Pietrzyk, Ewelina Baryla-Zapala, Małgorzata Gałązka-Sobotka, Iwona Kowalska-Bobko, Cezary Kępka, Tomasz Hryniewiecki

**Affiliations:** 1 II Department of Heart Arrhythmia, National Institute of Cardiology, Alpejska 42, 04-628 Warsaw, Poland; 2 Maple Health Group, 31-272 Cracow, Poland; krzysztof.andrzej.lach@gmail.com; 3 Institute of Public Health, Faculty of Health Science, Jagiellonian University Medical College, 31-008 Cracow, Poland; iw.kowalska@uj.edu.pl; 4 ASM Research Solutions Strategy, 99-300 Kutno, Poland; m.pietrzyk@asmresearch.pl (M.P.); e.zapala@asmresearch.pl (E.B.-Z.); 5 Institute of Healthcare Management, Lazarski University, 02-662 Warsaw, Poland; m.galazka-sobotka@lazarski.edu.pl; 6 Department of Interventional Cardiology and Angiology, National Institute of Cardiology, 04-628 Warsaw, Poland; ckepka@ikard.pl; 7 Department of Valvular Cardiac Defects, National Institute of Cardiology, 04-628 Warsaw, Poland; thryniewiecki@ikard.pl

**Keywords:** health technology assessment, hospital, innovation, hospital management

## Abstract

Hospital-based health technology assessment (HB-HTA) is a scientific approach to inform decisions on investments in health technologies across multiple medical specialties at a hospital level. HB-HTA is not currently practiced in Poland. This study aimed to assess the need for HTA in Polish hospitals, including perceived benefits and challenges of adoption of HB-HTA in Poland, expected demand for training in HB-HTA, and perception of incentives to foster HB-HTA adoption. Study data were gathered using the computer-assisted telephone interview (CATI) technique. Between June and August 2021, 50 interviews were conducted: 52% of respondents had over 10 years of experience, and 40% comprised the highest degree reference hospitals. A high or moderate need for HB-HTA was reported by 86% of managers. The ability to indicate valuable and affordable medical technologies was the main reported benefit of HB-HTA (90%). The main obstacle to the adoption of HB-HTA was the shortage of competent staff (84%). The most important incentives to adopt HB-HTA were free training and premium financing from the National Health Fund. There is a clear need for HB-HTA in Polish hospitals despite some important obstacles.

## 1. Introduction

According to 2019 estimates, there were over half a million medical technologies utilized in Europe on different healthcare levels, with hospitals being the key recipient thereof [1]. The growing interest in rational, scientifically sound, and impartial management of the inflow of innovative technologies has given birth to different approaches in supporting decisions on investments in health technologies. Health technology assessment (HTA), developed some five decades ago in the USA [2], was one of the approaches to that end, which gained substantial traction globally and is now a legally binding method of informing drug reimbursement decisions in multiple countries both at the national and regional level [3].

Deriving from this traditional HTA approach conducted at the national or regional level by dedicated organizations (e.g., Agency for Health Technology Assessment and Tariffs in Poland) is the hospital-based health technology assessment (HB-HTA). It is a scientific activity consisting of an in-depth comparative analysis of clinical, economic, financial, organizational, and other (societal, ethical, etc.) consequences of introducing new health technologies into hospitals [4]. Its unique characteristics compared with traditional HTA are the context of a specific hospital where the assessment takes place [5], types of information that hospital managers need (e.g., impact on hospital budget and organizational consequences bear more importance relative to other areas of assessment) [6], and types of technologies more frequently requiring an assessment in the hospital setting (non-drug technologies that are less frequently assessed by national HTA) [7]. The HB-HTA report is a document that includes the abovementioned information together with a recommendation to implement, or not, the assessed technology in a given hospital. HB-HTA applies to all types of health technologies, i.e., medicines, medical devices, imaging equipment, diagnostic tests, and even more complex organizational changes, such as introducing a new type of operating theater with ancillary care [7]. HB-HTA has been used in the past to inform decisions on investment in health technologies across multiple medical specialties, including oncology, cardiology, internal medicine, and others [7]. For example, an assessment process at a hospital level has been carried out for Barostim (baroreflex activation therapy) technology in the treatment of refractory hypertension to inform investment decisions in Spanish hospitals [8]. As demonstrated by studies, HB-HTA continues to bring value to hospital decision-makers in managing hospital formularies so that they include the most clinically and economically effective technologies while optimizing hospital budgets [4,9].

Since HB-HTA’s onset, its practices and activities have varied among hospitals; however, a research project AdHopHTA (Adopting Hospital-based Health Technology Assessment in the EU) carried out between the years 2012 and 2015 [10] consolidated the scattered HB-HTA knowledge and developed the best practices and tools, i.e., the AdHopHTA handbook [4] and toolkit [11], to help others embark on HTA in their hospitals. It continues to be a source of general guidance as to the development and optimization of HB-HTA activities. HTA at the hospital level is not currently practiced in Poland to the extent defined by the AdHopHTA handbook. Therefore, a research and implementation project, HB-HTA-PL, was initiated in 2019 to help introduce HTA into Polish hospitals [12].

The aim of the study was to assess the need for HB-HTA among managers of Polish hospitals, together with the main obstacles and incentives for adopting this approach.

## 2. Material and Methods

### 2.1. Study Questionnaire

A standardized questionnaire was developed to gather the following data: general information on the respondent and hospital, potential challenges for the implementation of HB-HTA in Polish hospitals, perceived benefits stemming from the adoption of HB-HTA, expected demand for training in HB-HTA, challenges in preparing an HB-HTA report, and the perception of incentives to adopt HB-HTA. The questionnaire was validated by five hospital managers before rolling it out to a full study sample. The managers agreed the questionnaire was comprehensive yet easy to understand, but the introduction to the interview was unnecessarily long. The questionnaire also covered topics concerning detailed information gathered during the preparation of an HB-HTA report; however, this material is not reported in this article.

### 2.2. The Interview

The research was carried out using the CATI technique, i.e., computer-assisted telephone interview, which is one of the empirical data collection techniques using CADAC (computer-assisted data collection) computer techniques. The CATI interview is a quantitative research technique that optimizes research in terms of time while maintaining a high quality of collected data [13]. The use of this technique is also justified in research conducted among hard-to-reach (mainly through direct contact) respondents [14]. CATI is probably a marginally inferior research approach compared to face-to-face or pen-and-paper personal interviews (PAPI), especially as far as sensitive or personal information is being gathered [15,16]. This was not the case in our study, and CATI was advantageous due to epidemic restrictions caused by the COVID-19 pandemic at the time of planning the study.

All interviews were conducted by two experienced pollsters who completed basic training in HB-HTA before the study.

The ASM Research Institute has a valid (as of 2021, 2022) PKJPA certificate, which guarantees that conducted research meets high quality (and ethical) standards. Moreover, the ASM Research Institute is a member of both the Polish Association of Public Opinion and Marketing Research Firms (OFBOR), whose statutory goal is to ensure high-quality research, and a member of the ESOMAR organization. The Institute operates in accordance with the standards set by the ESOMAR in the International Code of Marketing and Social Research Practice. Therefore, the study did not require the additional consent of the Bioethics Committee.

### 2.3. Respondent Sampling Procedure

A purposeful sampling was determined: the study covered 50 national clinical hospitals and large multi-specialist hospitals with cardiology or oncology departments. The sample size was fixed by the grant application and budget constraints. It was estimated based on the exploratory, innovative purpose of the research, expected interview duration, and recruitment of hard-to-reach respondents during the COVID-19 pandemic.

Due to the adopted research strategy, a restrictive procedure for recruiting potential respondents was assumed. First, an attempt was made to reach out to employees holding administrative or directorial positions in hospitals. In the case of non-response, employees holding managerial positions were invited. To ensure the reliability of the study and obtained data, interviews with respondents holding non-managerial positions were not planned. The interviews were conducted with representatives from the highest hospital referral levels, as well as primary and secondary referral hospitals. It was assumed that conducting interviews with respondents from these hospitals would be the most difficult and time-consuming. There were three attempts to contact the potential respondents; the interview was considered unfeasible only after the third unsuccessful attempt. A simplified diagram of this procedure is presented in the Supplementary Materials (Figure S1).

### 2.4. Statistical Analysis

Due to the size of the research sample, the authors decided to present the results of the study collectively (without presenting them by socio-demographic variables). Given the above, the following research results are presented in the tables and charts below, and each time refer to the entire study population, that is, 50 hospital representatives.

## 3. Results

The study was conducted between June and August 2021. As a result of the sampling procedure, a total of 402 phone calls were made (number of effective and ineffective calls to unique phone numbers). Overall, 41 calls had “no one answers/no such number” status (in the case of an inactive telephone number, the interview was construed as ineffective). There were 185 refusals to participate in the survey and 126 immediate disconnections: in this case, the respondent was not contacted again. The response rate (the ratio of effective interviews to the number of calls) to achieve 50 interviews was 12.4%, i.e., one in ten contacted hospital employees agreed to participate in the survey (Table S1). The mean time of the CATI interview was 42 min.

The baseline characteristics of the study group are summarized in Table 1. Ultimately, 20 interviews were conducted with representatives of the highest hospital referral level, and another 30 interviews were conducted among secondary and primary referral hospitals. Employees with administrative/directorial positions and managers comprised 78% and 22% of interviewed respondents, respectively. Half of the respondents had over 10 years of professional experience. All study respondents were experienced members of hospital senior management staff or managers designated for the interview by the general directorate of a hospital. Most centers were multi-profile hospitals; however, we also enrolled specialist hospitals, e.g., cardiology or oncology. About half of the respondents were already familiar with the term ‘HB-HTA’ before the interview (Table 1).

### 3.1. General Perception of the Need for HB-HTA in Poland

Altogether, 86% of respondents reported the need for implementation of HB-HTA in their hospitals: 32% reported a high need, and 54% reported a moderate need.

**Figure 1:**
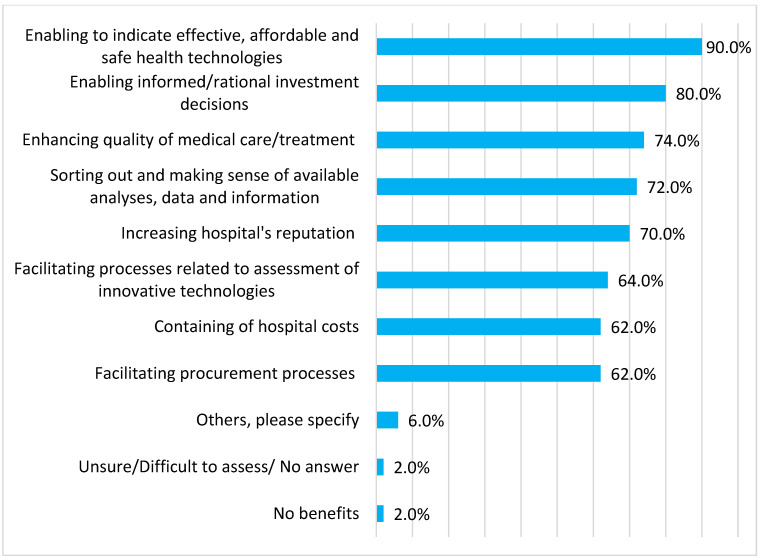
Opportunities and benefits of implementing health technology assessment in hospitals (HB-HTA).

Our respondents viewed HB-HTA mainly as a tool to select valuable and affordable medical technologies and to guide rational investment decisions in their hospitals (Figure 1). Of note, HB-HTA was also considered a means to increase a hospital’s reputation as an innovative and patient-orientated healthcare facility (Figure 1).

### 3.2. Obstacles to the Implementation of HB-HTA in Poland

**Figure 2:**
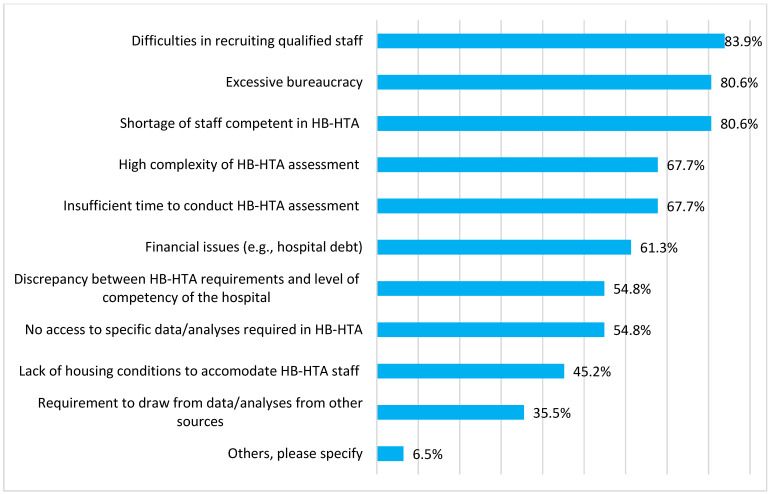
Main obstacles to the implementation of hospital-based health technology assessment (HB-HTA) in hospitals.

The majority of respondents (62%) identified problems with implementing HB-HTA in their hospitals, of which the most important one was the shortage of competent, well-qualified staff to conduct an HB-HTA (Figure 2 and Figure S2); 96% of surveyed managers reported at least a moderate need for HB-HTA training for their personnel. Other concerns expressed by hospital managers were potential complexity and high risk of excessive and unjustified bureaucracy related to technology assessment according to HB-HTA guidelines (Figure 2). As far as challenges to preparing an HB-HTA report in a respondent’s hospital were considered (Figure S3), again, the challenges of staff competency were the most prevalent (62%), followed by problems with access to relevant data (e.g., to conduct a review of literature, 58%) and fear of engaging in a generally time-consuming process (56%).

### 3.3. Potential Incentives for Polish Hospitals to Adopt HB-HTA

**Figure 3:**
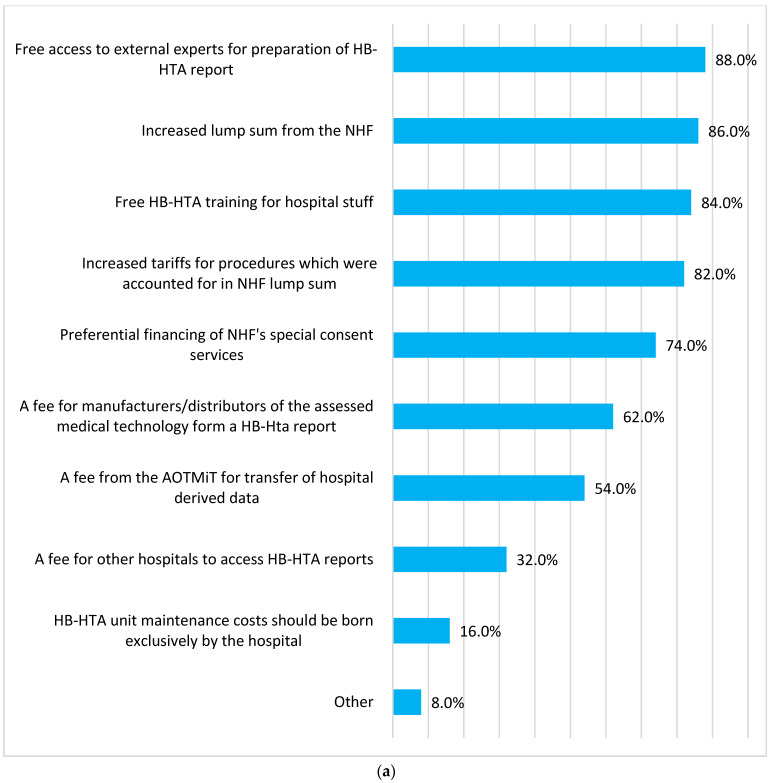

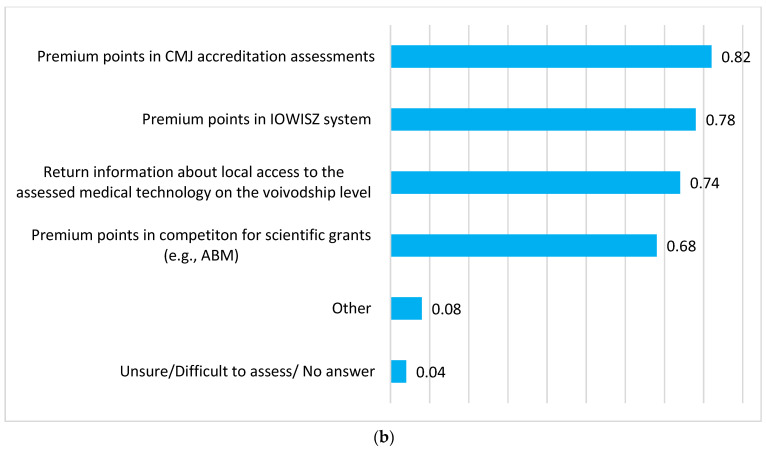
Financial (**a**) and non-financial (**b**) incentives for hospitals for adaptation health technology assessment (HB-HTA). NHF, National Health Fund. ABM, Medical Research Agency; CMJ, Centre for Monitoring Quality in Health Care; IOWISZ, Evaluation Instrument of Investment Motions in Health Care.

Figure 3 summarizes potential financial (a) and non-financial (b) incentives to adopt HB-HTA. Three financial incentives stood out. One was free access to training for local hospital staff or to external experts (Figure 3 and Figure S4). The other was an increase in the total lump sum that a hospital gets from the National Health Fund (NHF) or an increase in tariffs for certain procedures, which are accounted for in the lump sum. The third incentive was preferential financing of the NHF’s special consent services—a custom tariff for costly hospitalizations not covered by standard disease-related groups (DRGs) but calculated on a case-by-case basis. The most important non-financial incentive was an award of premium points during the accreditation process led by the Centre for Monitoring Quality in Health Care (CMJ).

## 4. Discussion

The study aimed to investigate the need to implement HTA in Polish hospitals, including the associated benefits and challenges, expected demand for training in HB-HTA, and perceived incentives to foster HB-HTA adoption in Poland. The main outcomes of this study conducted among 50 Polish hospital managers using the CATI methodology and associated questionnaire were: 1. 86% of respondents reported a high or moderate need for the implementation of HB-HTA in their hospitals; 2. The main perceived benefits of HB-HTA were the ability to indicate effective, affordable and safe health technologies and to make informed/rational investment decisions in health technology adoption; 3. The main obstacles to implementing HB-HTA were a shortage of competent staff and the risk of excessive bureaucracy; 4. The most important financial incentives to adopt HB-HTA were free access to training or external expertise and miscellaneous means to increase coverage for hospitalizations from the NHS; 5. The most important non-financial incentive was premium points during the hospital accreditation process.

To our knowledge, there are no published studies investigating the need for HB-HTA in countries where no HB-HTA activities are conducted. Conversely, various studies discuss HB-HTA experiences in the context of research areas investigated in our study. For instance, de Soárez et al. (2021) identified financial hurdles, those related to creating HB-HTA staff, and sustainability of HB-HTA operations as key challenges from the perspective of Brazilian hospitals [17]. All of these challenges were called out by the hospital managers we interviewed in our study. A 10-year survey of activities conducted at the HB-HTA unit in Lausanne, Switzerland (Centre Hospitalier Universitaire Vaudois) in 2016 found the overall usefulness of HB-HTA in improving the quality of investment decision-making [9]. Authors of the AdHopHTA handbook articulated it similarly as one of the reasons for implementing HB-HTA (“*HB-HTA provides hospital decision-makers (managers and clinicians) with science-based, multifaceted information and the necessary arguments on which to base the decision on whether or not to invest in a technology*”) [4]. In our survey, this was the highest-rated benefit of implementing HB-HTA in Poland (enabling us to indicate effective, affordable and safe health technologies selected by 90% of hospital managers). HB-HTA as a means of improving the efficiency of managing hospital budgets was another reason for adopting HB-HTA called out by authors of the AdHopHTA handbook [4], and it was seen as a key benefit by 62% of hospital managers we interviewed (“*containing hospital costs*”). Both examples show that the expectations of Polish hospital managers as to the potential benefits of adopting HB-HTA are in line with the reasons for implementing HB-HTA articulated in the AdHopHTA handbook [4]. Moreover, in 2015, researchers of the AdHopHTA project proposed guiding principles for good practices in HB-HTA units that are based on extensive research and experiences of mature HB-HTA units in Europe [18]. These guiding principles resonate with the findings of our study; for example, in guiding principle 12, AdHopHTA research stresses the importance of having well-defined unit personnel and ad hoc experts. This principle is mirrored in the findings of our research, both as obstacles and incentives. Our surveyed managers indicated difficulties in recruiting qualified staff as the most prevalent obstacle (84% of respondents), and the free access to external experts appeared as an important incentive for implementing HB-HTA [18]. Cicchetti et al. (2017), in another publication resulting from the AdHopHTA project, pointed out that formalization/structuration is an important factor in setting up HB-HTA practices [19]. This corresponds well with the excessive bureaucracy as the second most prevalent obstacle expressed by our study participants. Other experiences with HB-HTA, i.e., IMPlementation of A Quick hospital-based HTA (IMPAQHTA) in the Lombardy Region (Italy) in 2017, have shown that allowing for a rapid assessment and the provision of relevant information that hospital managers need for making investment decisions in health technologies are some of the key features of a wholesome and implementable HB-HTA framework [20]. Stadig et al. (2021) also approached the issue of the availability of relevant information by describing the practices of data retrieval, underlining the involvement of librarians [21]. These elements were reflected in our research as benefits (sorting out and making sense of available data/information) and obstacles (insufficient time to conduct an HB-HTA assessment). Overall, findings from the studies discussing current HB-HTA practices of experienced units around the world indicate similar challenges and opportunities that Polish hospitals unfamiliar with HB-HTA would expect.

Our respondents identified a number of benefits of implementing HB-HTA in their hospitals. As expected, HB-HTA was considered a tool to indicate valuable and affordable medical technologies and to guide rational investment decisions in their hospitals. This is the first and foremost aim of HB-HTA. Managers also indicated its applicability to improve the quality of medical care delivered in the hospital and, therefore, the hospital’s reputation. Only one respondent did not identify any benefits from implementing HB-HTA.

The main obstacle to implementing HB-HTA in Poland was the shortage of qualified staff trained in HB-HTA. This was evident from questions concerning obstacles and incentives. Respondents clearly stated that they lack the proper staff and expected to benefit from free training and support from external experts. Another important obstacle was the potential complexity of an HB-HTA report, which might not reflect the actual perspective of the hospital and fear of unjustified bureaucracy. As stated before, HB-HTA, by definition, adopts a hospital perspective, and therefore, those latter issues are largely unjustified [4,5].

Identified incentives for adopting HB-HTA in a hospital can be broadly divided into two categories. The first comprised access to free training and support from experts. The second comprised various opportunities to increase the amount of funding received from the NHF. It was closely related to the Polish system of financing hospital services, including lump sums, DRGs and special consent services [22]. Overall, our respondents would expect to get a premium to the hospital lump sum, better tariffs for DRGs or preferential treatment when applying for special consent services. Of particular interest is that the non-financial incentives on which we interviewed the respondents showed that, ultimately, financial gratification was important. For example, the main reasons hospital go through the CMJ accreditation process is to get a premium on their lump sum.

## 5. Limitations

Our research was exploratory and innovative, as it was the first such study in Poland. This was associated with numerous challenges, the most important of which was to develop a questionnaire that maximizes the probability of obtaining reliable information and limits the possibility of terminated interviews (which is often caused by the respondents’ impatience related to the excessive length of a questionnaire). Our questionnaire was a rather extensive tool (the interview lasted 40 min on average); thus, the main anticipated risk was the high percentage of terminated (ineffective) interviews. An additional challenge was to recruit hard-to-reach respondents with a complex configuration of features during the COVID-19 pandemic. This research strategy, although essential, limited the availability of the respondents (the possibility of obtaining direct contact with the selected respondent). Given the above research challenges, the determined sample size (50 hospital representatives) turned out to be sufficient. From the methodological point of view, theoretical saturation was reached, confirming the positive verification of the test result. The theoretical saturation means that the empirical, collected data fully reflected the properties of the researched sample, and thus, a certain repeatability of the respondents’ answers was observed [23,24].

## 6. Conclusions

Our study has shown that Polish managers need HB-HTA in their hospitals to make informed decisions on the adoption of effective, affordable and safe health technologies. There are several local and systemic pre-requisites and incentives for the successful implementation of HB-HTA in Polish hospitals, i.e., ensuring the availability of competent staff and avoiding risks of excessive bureaucracy related to the HB-HTA process. Moreover, free access to training or external expertise and incorporating mechanisms to increase coverage for hospitalizations from the NHS were viewed as key financial incentives to foster the adoption of HB-HTA by hospitals. Finally, Polish hospital managers viewed premium points during the hospital accreditation process as another key non-financial incentive to implement HB-HTA in their hospitals.

## Figures and Tables

**Table 1:** Baseline characteristics of the study group.

	Number of Respondents *n* (%)
Type of hospital	
General hospital	42 (84%)
Specialized cardiology center	1 (2%)
Specialized oncology center	3 (3%)
Other	4 (8%)
Respondent’s position	
Director general/member of the executive board director	39 (78%)
Designated manager	11 (22%)
Other	0 (0%)
Work experience	
Less than 1 year	1 (2%)
1–3 years	16 (32%)
4–6 years	4 (8%)
7–9 years	3 (6%)
More than 10 years	26 (52%)
Previous knowledge of HB-HTA	
Yes	23 (46%)
No	27 (54%)

## Data Availability

The data presented in this study are available on reasonable request from the corresponding author.

## Supplementary Materials

The following supporting information can be downloaded at: https://www.mdpi.com/article/10.3390/ijerph19148855/s1, Figure S1: Study recruitment procedure; Figure S2: Availability of staff competent in HB-HTA in respondent’s center; Figure S3: Challenges in preparing a hospital-based health technology assessment (HB-HTA) report in respondent’s hospital; Figure S4: The reported need for training in HB-HTA; Table S1: Study recruitment statistics.
