# Supplementary material for: The Need to Implement Health Technology Assessment in Polish Hospitals—A Survey of 50 Hospital Managers
Source: Int J Environ Res Public Health. 2022 Jul 21;19(14):8855. doi: 10.3390/ijerph19148855 (PMC9319323; doi:10.3390/ijerph19148855)
Supplement: Supplementary file 1 [file ijerph-19-08855-s001.zip › ijerph-1799230-supplementary.pdf]

Supplementary Materials

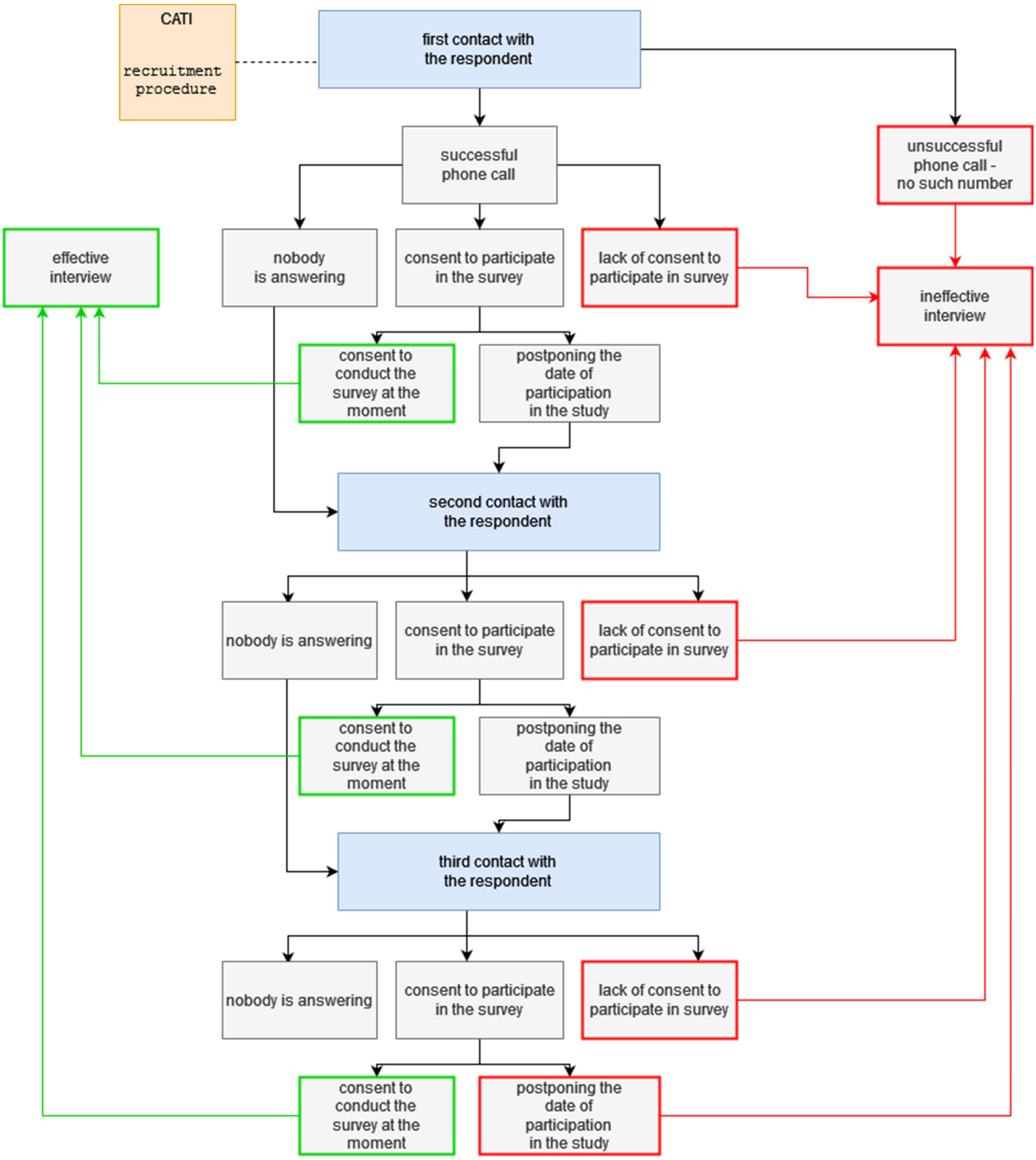

Figure S1. Study recruitment procedure.

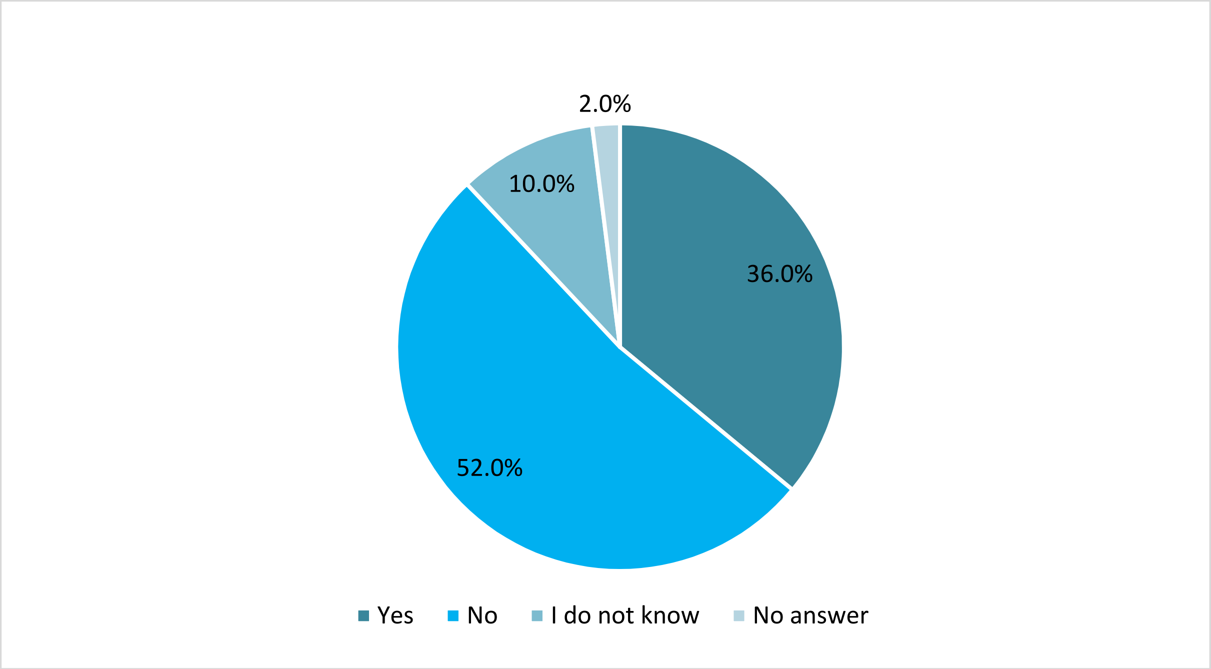

Figure S2. Availability of staff competent in HB-HTA in the respondent's center.

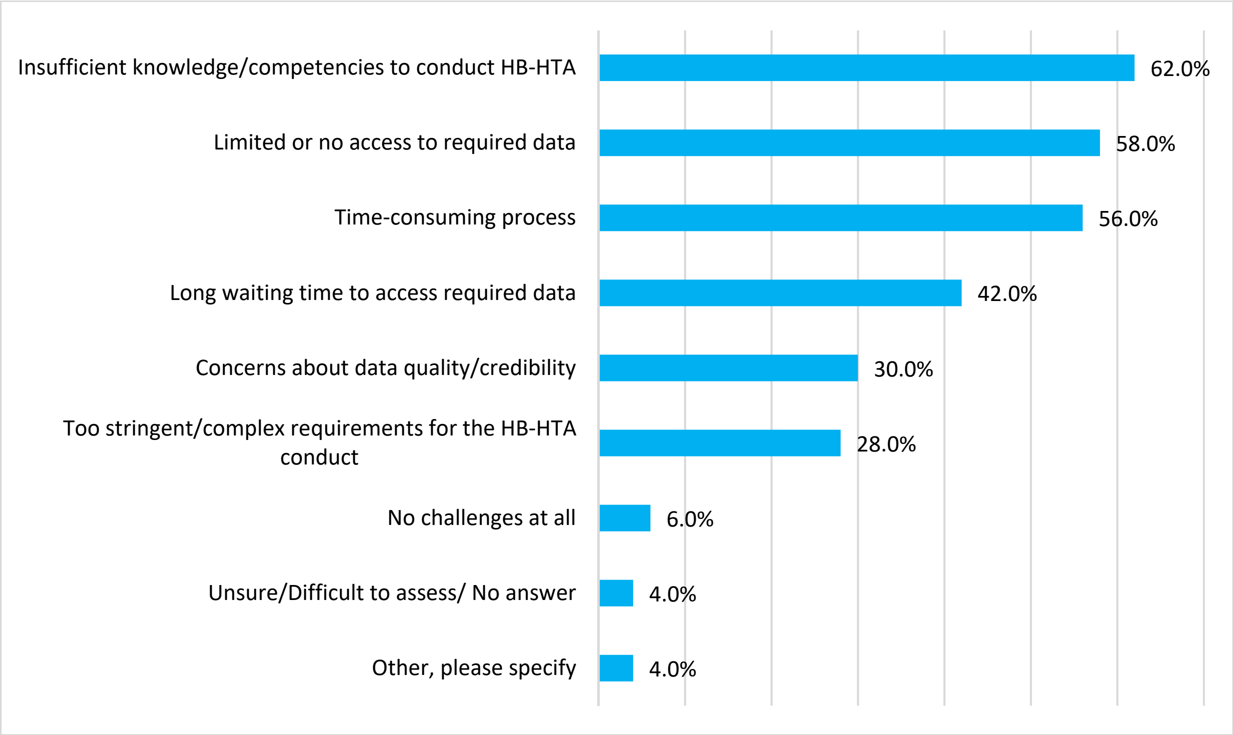

Figure S3. Challenges in preparing a hospital-based health technology assessment (HB-HTA) report in the respondent's hospital.

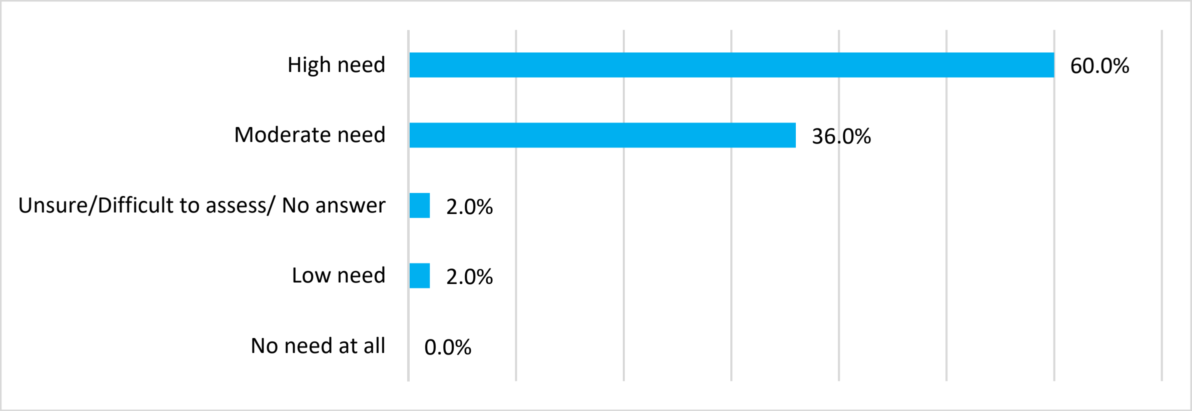

Figure S4. The reported need for training in HB-HTA for the respondent's employees.

Table S1. Study recruitment statistics.

**Data**	**Amount**	**Percent**
Number of subjects in database	800	100.0%
Number of all calls made (effective and ineffective)	402	50.2%
Effective interviews	50	6.3%
**CATI Status**	**Amount**	**Percent**
No answer/no such number	41	10.2%
Refusal	185	46.0%
Immediate disconnection	126	31.3%
Consent/effective interview	50	12.4%
Total	**402**	**100%**
